# Added Sugars in School Meals and the Diets of School-Age Children

**DOI:** 10.3390/nu13020471

**Published:** 2021-01-30

**Authors:** Mary Kay Fox, Elizabeth C. Gearan, Colin Schwartz

**Affiliations:** 1 Mathematica, 955 Massachusetts Avenue, Suite 801, Cambridge, MA 02139, USA; lgearan@mathematica-mpr.com; 2 Center for Science in the Public Interest, 1220 L Street NW, Suite 300, Washington, DC 20005, USA; cschwartz@cspinet.org

**Keywords:** added sugars, Dietary Guidelines for Americans, National School Lunch Program, School Breakfast Program, school meals, nutrition standards, school-age children, dietary intake, School Nutrition and Meal Cost Study

## Abstract

Research is limited on added sugars in school meals and children’s dietary intakes after the 2015–2020 Dietary Guidelines for Americans (DGA) recommended that added sugars be limited to less than 10% of total calories. This analysis uses data from the School Nutrition and Meal Cost Study (SNMCS) to examine levels of added sugars in: (1) school meals and (2) children’s dietary intakes at breakfast, lunch, and over 24 h on school days. SNMCS data were collected in the 2014–2015 school year after updated nutrition standards for school meals were implemented. Most schools exceeded the DGA limit for added sugars at breakfast (92%), while 69% exceeded the limit at lunch. The leading source of added sugars in school meals (both breakfasts and lunches) was flavored skim milk. More than 62% of children consumed breakfasts that exceeded the DGA limit, and almost half (47%) consumed lunches that exceeded the limit. Leading sources of added sugars in the breakfasts consumed by children were sweetened cold cereals and condiments and toppings; leading sources of added sugars in children’s lunches were flavored skim milk and cake. Over 24 h, 63% of children exceeded the DGA limit. These findings show that school meals and children’s dietary intakes are high in added sugars relative to the DGA limit and provide insights into the types of foods that should be targeted in order to decrease levels of added sugars.

## 1. Introduction

Overconsumption of added sugars has been identified as an important public health concern [1,2,3]. Among children, intake of added sugars has been associated with increased weight gain/adiposity [4,5,6,7,8], poor diet quality [9], dental caries [10,11], and increased risk of cardiovascular disease [12,13,14,15]. Added sugars, which provide calories but have no nutritional benefit, include sugars, syrups, or caloric sweeteners that are added to foods and beverages during processing, food preparation (at home, restaurants, or other places), or at the table [16]. Since 2003, the World Health Organization has recommended that intake of added sugars be limited to less than 10% of total calorie intake [17]. The Dietary Guidelines for Americans (DGA) have long recommended limiting intake of added sugars but did not set a specific standard until 2015. The 2015–2020 DGA recommend that intake of added sugars be limited to less than 10% of total calories per day [2]. Evidence shows that the diets of most school-age children (6 to 19 years of age) in the United States (U.S.) are not consistent with this recommendation [18]. 

The potential for schools to positively influence children’s diets has long been recognized. Almost all public schools in the U.S. participate in the school meal programs—the National School Lunch Program (NSLP) and the School Breakfast Program (SBP) [19,20]. On an average school day in 2019, 29.6 million children, or about half of the student population, ate a school lunch and 14.8 million children ate a school breakfast [21,22]. Among children who consume both a school breakfast and a school lunch, school meals account for nearly half of their daily calorie intake [23,24]. The school meal programs are especially important for low-income children who are eligible to receive meals free or at a reduced-price (FRP). In 2019, 74% of lunches and 85% of breakfasts were served FRP [21,22]. 

School meals are required to meet nutrition standards that specify the types and amounts of foods to be offered. In 2010, Congress passed the Healthy, Hunger-Free Kids Act (HHFKA, Public Law 111–296) that mandated changes in the nutrition standards to improve alignment of school meals with the DGA and improve the overall nutritional quality of the meals. In 2012, the U.S. Department of Agriculture (USDA), which administers the school meal programs, published the updated standards [25]. The standards specified age-based minimum and maximum standards for calories, reduced sodium levels, and eliminated synthetic forms of trans fat. They also limited milk to low-fat and nonfat varieties and required inclusion of whole grain-rich foods, a wider range of vegetables, larger portions of fruits and vegetables, and free drinking water. Finally, the updated standards added a requirement that students select at least 1/2 cup of fruits or vegetables in order for their meal to be eligible for Federal reimbursement. The updated standards did not specifically limit levels of added sugars in school meals; however, they restricted flavored milk to nonfat milk only and limited the number of grain-based desserts that could be offered at lunch each week. In addition, it was expected that planning menus to meet (not exceed) the maximum calorie limits would constrain use of foods high in added sugars [25]. The updated standards were phased in over several years, beginning in school year (SY) 2012–2013; school were required to meet all requirements for both breakfasts and lunches by SY 2014–2015 [25]. 

Research has shown that the nutritional quality of school meals and children’s intakes from school meals has improved since implementation of the updated nutrition standards [26,27,28,29,30]. However, most of these studies assessed nutritional quality using the Healthy Eating Index (HEI)-2010, which was designed to assess alignment with the 2010 DGA [31,32]. Because the 2010 DGA did not include a specific recommendation for added sugars [33], the HEI-2010 does not include a separate component that assesses added sugars. Rather, the index includes an “empty calories” component that assesses compliance with recommended limits for empty calories from solid fats, added sugars, and alcohol collectively [31,32]. The one study that did separately examine added sugars found no association between consuming school meals and children’s intake of added sugars [30]. Given that the 2015–2020 DGA include a quantitative limit for calories from added sugars, it is important to assess levels of added sugars in school meals and in children’s dietary intakes at school. This paper is intended to fill these important gaps in the knowledge base about school meals and the diets of school-age children. The analysis uses data from the School Nutrition and Meal Cost Study (SNMCS), a comprehensive national study of the school meal programs that collected data two school years after the updated nutrition standards were implemented, to directly examine levels of added sugars in school meals and children’s diets. The analysis also assesses variation in key outcomes across subgroups of schools and children and identifies leading sources of added sugars. 

## 2. Materials and Methods 

### 2.1. Study Design, Samples, and Primary Data Sources 

The SNMCS included nationally representative samples of public-school food authorities (SFAs) in the 48 contiguous states and the District of Columbia that participated in the NSLP, non-charter schools within these SFAs, and students who attended these schools. Data were collected during the spring semester of SY 2014–2015. This analysis uses two primary data sources: (1) school menu data to examine levels of added sugars in school meals, and (2) 24-h dietary recall data to examine levels of added sugars in children’s dietary intakes. 

School menu data were provided by school nutrition managers (or their designee), who completed an online menu survey for one school week (referred to as the target week). The menu survey collected detailed information about all foods and beverages offered in reimbursable breakfasts and lunches on each day of the target week. This included, for each menu item, detailed food descriptions, portion sizes, recipes and the number of portions prepared. Detailed instructions for completing the menu survey were embedded in the instrument. In addition, online training videos were provided and trained technical assistants checked in with respondents before and during the target week. Respondents who were unable or unwilling to complete the online instrument were offered the option of completing a paper-and-pencil version. The weighted response rate for the school menu data was 96.2% [34]. The final analysis sample included 1207 schools with complete data for school lunches and 1111 schools with complete data for school breakfasts.

Data on children’s dietary intakes were collected through 24-h dietary recall interviews. In schools that completed the menu survey, 24-h dietary recalls were completed with 2165 students 6 to 19 years of age (weighted response rate of 63.6% [24]). The dietary recalls were administered by trained interviewers using USDA’s Automated Multiple Pass Method [35] and collected detailed information on all foods and beverages consumed over 24 h on a school day. Middle and high school students completed the dietary recall in one interview, reporting on the previous day’s intake. Elementary school students completed the dietary recall in two separate interviews. The first interview was conducted as soon as possible after students’ lunch periods and collected information about foods and beverages consumed from waking through lunch. The second interview was conducted with parental assistance, usually the following day, and collected information about foods and beverages consumed the rest of the 24-h period.

Both the menu survey and dietary recall data included nutrient values from the Food and Nutrient Database for Dietary Studies, version 2011–2012 [36] for each reported food and beverage, as well as amounts of USDA Food Pattern food groups from the Food Patterns Equivalents Database, version 2011–2012 [37]. In the dietary recall data, the study identified foods that children consumed for breakfast and lunch (separately) using established procedures that considered eating occasions reported by children and the time of day foods were consumed [38,39]. Foods identified as breakfast and lunch foods included all foods reported for the meal, including a foods obtained from school meals, foods from home (breakfasts consumed at or brough from home and lunches brought from home), and other foods children may have obtained at school or from other sources. For foods that students obtained from school meals, the study incorporated nutrient and food group values from the menu survey to ensure that the nutrient and food group content of these foods were accurately represented in students’ dietary intake data. 

The Office of Management and Budget and the New England Institutional Review Board (NEIRB) approved the protocol for the SNMCS (NEIRB# 14-344). The study also followed any institutional review processes required by specific school districts. Passive or active consent (depending on the local school district’s requirements) was obtained from parents and students before collecting the 24-h dietary recall data. The methodology report for the SNMCS describes in detail the study’s design, as well as sampling, recruitment, data collection, and data processing procedures [40]. Additional details about the collection and analysis of data used in this paper are available in Volumes 2 and 4 of the SNMCS final report [24,34].

### 2.2. Data on School and Child Characteristics

The analysis examined variation in the added sugar content of school meals across subgroups of schools defined by school level (elementary, middle, and high schools), poverty level, and racial and ethnic composition. Data on poverty level and racial and ethnic composition came from the U.S. Department of Education’s Common Core of Data (CCD) Local Education Agency (School District) Universe Survey for 2011–2012 [41]. The CCD defines schools with 25.0% or fewer students eligible for FRP lunches as low poverty; schools with 25.1% to 50.0% as mid-low poverty; schools with 50.1% to 75.0% as mid-high poverty; and schools with more than 75.0% as high poverty. Data on schools’ racial and ethnic composition also came from the 2011–2012 CCD. It classified schools into one of four subgroups based on the racial and ethnic composition of students who attended the school: majority White; majority Black; majority Hispanic; and diverse. Schools in which two-thirds of students identified as non-Hispanic White were classified as majority White schools, whereas schools in which 50% or more of students identified as non-Hispanic Black were classified as majority Black. Similarly, schools in which 50% or more students identified as Hispanic were classified as majority Hispanic. Schools that did not fit into any of these categories were classified as diverse [42]. Nine schools that were missing information on the racial and ethnic composition of students were omitted from analyses that explored differences by race/ethnicity subgroups. 

The analysis also examined variation in intakes of added sugar across subgroups of children defined by school level, gender, eligibility for FRP school meals, race and ethnicity, participation in the NSLP, and participation in the SBP. Data on eligibility for FRP meals and race and ethnicity were obtained from the study’s parent interview. All of the other child characteristics were available on the 24-h recall file. The SNMCS used school administrative data to identify students who participated in the NSLP and SBP on the day covered in their 24-h recall. When administrative data were not available (13% of students at breakfast and 9% at lunch), the study imputed participation status using established procedures that considered the types of foods students consumed and where the foods were obtained [38,39]. Subjects who were missing information for any of the child characteristics were excluded from the respective analysis.

### 2.3. Estimating Levels of Added Sugars in School Meals and Children’s Dietary Intakes and Compliance with DGA Limit 

The analysis examined the level of added sugars in school meals by estimating the mean calories from added sugars and the mean percentage of total calories from added sugars. The SNMCS menu survey data included school-level variables for (1) the average number of total calories and (2) the average number of calories from added sugars in breakfasts and lunches prepared in each school during the target week. These two variables, which reflect the “weekly average” amounts of total calories and calories from added sugars in school breakfasts and lunches, were constructed by the SNMCS study team using a four-step process [34]. First, for each daily breakfast or lunch menu, the team multiplied the amount of added sugars in one portion of each food by the number of portions of the food that were prepared that day. Second, these values were summed across all foods offered on the daily menu. Third, these daily menu totals were divided by the number of breakfasts or lunches prepared that day. Finally, these daily averages were averaged across all menu days in the school to get the weekly average amount of added sugars in breakfasts and lunches prepared in each school. The resulting estimates of added sugar content give greater weight to menu items that were prepared in larger quantities, which generally reflects the popularity of the various menu items among children. 

Data on the average number of total calories and the average number of calories from added sugars were used to estimate the average percentage of total calories provided by added sugars in each type of meal in each school:

*Percentage of total calories from added sugars* = (*calories from added sugars ÷ total calories*) × 100.

The analysis then compared the average percentage of total calories from added sugars in breakfasts and lunches in each school to the recommended DGA limit of less than 10% to estimate the percentage of schools that exceeded the DGA limit. Outcomes were estimated for all schools combined and by subgroup. 

Comparable analyses were conducted to describe children’s intakes of added sugars on school days using the 24-h recall data. Separate analyses were conducted to examine children’s intakes of added sugars at breakfast, lunch, and over 24 h. Outcomes were estimated for all children combined and by subgroup. Children who did not consume a breakfast or lunch on the day covered in the 24-h recall were excluded from the associated meal-specific analysis. All children were included in the analysis of 24-h intakes, regardless of whether they consumed breakfast or lunch. 

### 2.4. Identifying Leading Sources of Added Sugars

To identify leading sources of added sugars in school meals and in children’s diets, the analysis used a food grouping scheme developed by SNMCS researchers to classify foods reported in school meals and dietary recalls into major and minor food groups. To simplify the presentation of findings, some minor food groups were combined, following the approach SNMCS researchers used in a comparable analysis [34], to create a streamlined set of food groups. To estimate the relative contribution of different major and minor food groups to added sugars in school meals, the analysis first summed the amount of calories from added sugars provided by a given food group across all meals prepared by schools and then divided by the total amount of calories from added sugars provided in all meals prepared by schools. Comparable analyses were conducted to examine leading sources of added sugars in the lunches and breakfasts consumed by children. 

In interpreting findings from this type of analysis, it is important to recognize that the relative contribution of a food or food group as a source of added sugars is determined by two things—(1) the amount of added sugars in the food and (2) the frequency with which the food was offered (in school meals) or consumed (by children) [43]. For this reason, foods that are more commonly offered in school meals or consumed by children may make more substantial contributions to total amounts of added sugars than might be expected based on added sugars content alone [43]. In addition, it is important to understand that not all of the children in the study consumed school meals—some children consumed meals obtained from home or from other places. For this reason, it is not expected that leading sources of added sugars in the meals consumed by children would necessarily align with the leading sources of added sugars in school meals. 

### 2.5. Statistical Methods 

Descriptive analyses were conducted as described in Section 2.3 and Section 2.4. All calculations were performed in SAS, version 9.4 and R, version 4.0.3, and all analyses were weighted using sampling weights that accounted for differences between the study sample and the reference population, the study’s complex sample design, and nonresponse. Analyses of the school menu survey data used school-level weights, and analyses of the 24-h recall data used separate student-level weights. The weights were designed to bring the weighted distributions of the school and student samples in line with the corresponding population distributions and reduce, to the greatest extent possible, the potential for bias resulting from the sampling design or nonresponse [40]. 

For the primary analytic outcome—the percentages of schools and children exceeding the DGA limit for added sugars—the statistical significance of differences between subgroups of schools and students (*p* < 0.05) was assessed using two-tailed *t*-tests. Independent *t*-tests were conducted for all potential comparisons within a subgroup. For example, for subgroups of schools and students defined by school level, differences were tested for three comparisons: elementary versus middle, elementary versus high, and middle versus high. 

## 3. Results

### 3.1. Added Sugars in School Meals 

School breakfasts prepared in SY 2014–2015 provided an average of 88 calories from added sugars (Table 1). On average, added sugars accounted for 17% of calories in school breakfasts, a level that is 70% higher than the 2015–2020 DGA limit of less than 10%. More than nine in ten schools (92%) prepared breakfasts that exceeded the DGA limit. There were no significant differences between elementary, middle, and high schools in the percentage of schools that exceeded the DGA limit for added sugars. However, low poverty and mid-low poverty schools were significantly more likely than high poverty schools to exceed the DGA limit (95% for both groups versus 87%). In addition, schools with a majority White student body were significantly more likely than majority Hispanic or diverse schools to exceed the DGA limit (97% versus 84% and 89%, respectively). 

School lunches prepared in SY 2014–2015 provided less added sugar, relative to school breakfasts, and were more likely than school breakfasts to meet the DGA limit (Table 1; differences between breakfasts and lunches were not tested for statistical significance). On average, school lunches provided 75 calories from added sugars, which accounted for 11% of calories in school lunches. This average was close to the DGA limit; however, more than two-thirds of schools (69%) prepared lunches that exceeded the DGA limit. There were no significant differences in the likelihood of school lunches meeting the DGA limit for added sugars across subgroups of schools with different levels of poverty or different racial/ethnic compositions. However, there were notable differences across school levels. Elementary schools were more likely than either middle schools or high schools to exceed the DGA limit for added sugars (75% versus 66% and 57%, respectively), and middle schools were more likely than high schools to exceed the DGA limit (66% versus 57%).

**Sources of Added Sugars.** In both breakfasts and lunches, the leading source of added sugars was flavored skim milk (Table 2). Flavored skim milk contributed 29% of the added sugars in school breakfasts and almost half (47%) of the added sugars in school lunches. Other leading sources of added sugars in school breakfasts included sweetened cold cereals (13%), condiments and toppings (which includes syrup, jelly, and jam; 12%), muffins and sweet/quick breads (7%), and granola and breakfast bars (5%). The only other food group to contribute more than 5% of added sugars in school lunches was condiments and toppings (9%). Flavored 1% milk and breads, rolls, bagels and other plain breads each contributed 3% of the added sugars in school lunches. 

### 3.2. School-Age Children’s Intakes of Added Sugars at Breakfast and Lunch 

In SY 2014–2015, breakfasts consumed by school-age children provided an average of 63 calories from added sugars (Table 3). On average, added sugars accounted for 16% of calories in children’s breakfasts, which is 60% higher than the 2015–2020 DGA limit. The breakfasts consumed by more than six in ten children (62%) exceeded the DGA limit. There were no significant differences in the likelihood of children consuming a breakfast that exceeded the DGA limit for added sugars among subgroups defined by school level, gender, eligibility for FRP meals, or participation in the NSLP or SBP. However, non-Hispanic White children, non-Hispanic Black children, and Hispanic children were all significantly more likely than children in the multiracial/other group to consume breakfasts that exceeded the DGA limit (63 to 64% versus 47%).

On average, lunches consumed by school-age children in SY 2014–2015 provided 67 calories from added sugars and 11% of all lunch calories from added sugar (Table 3). Relative to breakfasts, the percentage of calories from added sugars in children’s lunches was closer to the DGA limit of less than 10% of calories (the statistical significance of differences between children’s breakfasts and lunches was not tested). Nonetheless, close to half (47%) of school-age children consumed lunches that exceeded the DGA limit for added sugars. There were no significant differences in the likelihood of children consuming a lunch that exceeded the DGA limit for added sugars among subgroups defined by school level, eligibility for FRP meals, or participation in the NSLP or SBP; however, there were some significant differences across gender and race/ethnicity subgroups. Specifically, females were significantly less likely than males to consume a lunch that exceeded the DGA limit for added sugars (44% versus 50%), and non-Hispanic White children were significantly more likely than Hispanic children or children in the multiracial/other group to consume lunches that exceeded the DGA limit (51% versus 44% and 42%, respectively).

**Sources of Added Sugars.** Assessment of the leading sources of added sugars in the breakfasts and lunches consumed by school-age children considered all foods children consumed at each meal. This included foods provided by school meals, foods from home (breakfasts consumed at or brought from home and lunches brought from home), and other foods children may have obtained at school or from other sources. The two leading sources of added sugars in children’s breakfasts were sweetened cold cereals, which contributed 23% of all added sugars, and condiments and toppings (13%) (Table 4). Toaster pastries and granola bars each contributed 5% of added sugars and muffins and sweet/quick breads contributed 4%. Several beverages were leading contributors to added sugars at breakfast—(sweetened) tea and coffee, fruit drinks, flavored skim milk, and carbonated sodas each contributed approximately 4% of the added sugars in children’s breakfasts. Yogurt was the tenth leading contributor to added sugar intakes at breakfast (3%). 

The leading sources of added sugars in lunches consumed by school-age children were flavored skim milk (16%) and cake (11%) (Table 4). Peanut butter sandwiches contributed 7% of added sugars and candy, fruit drinks, and condiments and toppings each contributed 6%. Other foods included in the top ten contributors to added sugar intakes at lunch included carbonated sodas (5%), sports and energy drinks (4%), (sweetened) tea and coffee (4%), and granola bars and breakfast bars (4%).

### 3.3. Children’s Intakes of Added Sugars over 24 Hours

Children’s dietary intakes over 24 h were not consistent with the DGA recommended limit for added sugars. On average, 13% of the calories in the 24-h intakes of school-age children came from added sugars, and the 24-h intakes of almost two-thirds (63%) of children exceeded the DGA limit of less than 10% (Table 5). There were no significant differences in the likelihood of exceeding the DGA limit among subgroups of children defined by school level, gender, eligibility for FRP meals, or participation in the NSLP or SBP. However, both non-Hispanic White children and non-Hispanic Black children were significantly more likely than Hispanic children or children in the multiracial/other group to exceed the DGA limit (67% and 71% for non-Hispanic Whites and non-Hispanic Blacks, respectively, versus 59% and 52% for Hispanics and multiracial/other). 

## 4. Discussion

This analysis shows that school lunches and breakfasts are high in added sugars, relative to the 2015–2020 DGA recommendation. The analysis also found that the dietary intakes of school-age children at breakfast, lunch, and over 24 h were high in added sugars. Existing nutrition standards for school meals do not include a standard for added sugar content. However, the overarching goal of the standards is to align school meals with the DGA and ensure their nutritional quality. Although research has shown that the nutritional quality of school meals has improved since updated nutrition standards were implemented starting in SY 2012–2013 [24], the levels of added sugars documented in this analysis are cause for concern. 

Levels of added sugars in school breakfasts are of particular concern, given that breakfasts prepared in 92% of all schools exceeded the DGA limit. While the problem is widespread, results showed that breakfasts prepared in low poverty, mid-low poverty, and majority White schools were more likely to exceed the DGA limit for added sugars than other types of schools. This pattern is consistent with another analysis of SNMCS data which documented variation in the healthfulness of school food environments by poverty level and racial ethnic composition [44]. Levels of added sugars in school lunches are of particular concern for elementary schools. Lunches in three-quarters of elementary schools exceeded the DGA limit versus 66% of middle schools and 57% of high schools. This disparity is associated with the fact that the nutrition standards specify calorie ranges that reflect the calorie requirements of children of different ages. For school lunches, calorie ranges are 550–650 for children in kindergarten through grade 5; 600–700 for children in grades 6–8; and 750–850 for children in grades 9–12 [25]. Thus, in absolute terms, lunches prepared in elementary schools have less room for empty calories from added sugars. With current levels of added sugars, it may be hard for elementary schools to prepare lunches that do not exceed the maximum calorie level. Indeed, the SNMCS found that higher proportions of elementary schools exceeded the maximum calorie level for NSLP lunches (40%) than either middle schools (34%) or high schools (14%) [34]. 

Leading contributors to added sugars in school meals include flavored milks, sweetened cold cereals, condiments and toppings, and, particularly for breakfasts, sweet bakery products. This pattern is generally consistent with findings from analyses of data from the What We Eat in America/National Health and Nutrition Examination Survey [18], with the exception of sugar-sweetened beverages, which are not permitted in school meals. In addition to establishing a quantitative standard for added sugars in school meals, USDA may consider establishing limits on sugar content of ready-to-eat cereals and bakery products, similar to the limit on sugar content of cereals included in food packages for the Special Supplemental Nutrition Program for Women, Infants, and Children [45]. Another strategy may be to limit the frequency of these foods in planned menus as well as foods that are offered with sweetened toppings. The new requirement for Nutrition Facts labels to list amounts of added sugars will allow schools to more easily track the added sugars content of foods when planning menus [46]. 

Given the large contribution flavored skim milk made to added sugars in both school breakfasts and lunches, USDA may also want to consider limiting flavored milk—for example, allowing flavored milks at one meal but not both— or limiting how often flavored milk can be offered in a week. When SNMCS data were collected in SY 2014–2015, the updated nutrition standards specified that only skim milk could be offered in flavored varieties, and most schools adhered to this requirement [34]. However, starting in 2019, USDA has allowed schools to offer both skim and low-fat flavored milks [47], which has the potential to increase the already high levels of added sugars in school meals and, as a result, children’s dietary intakes. Several stakeholders support the recent flexibility USDA has given schools related to flavored low-fat milk because of the need to improve calcium intakes and bone health in school-age children; however, they acknowledge that increased calories from flavored low-fat milk needs to be offset by other menu changes to ensure that calorie maximums are not exceeded [48,49]. 

Promoting water consumption may also decrease children’s consumption of added sugars from sweetened beverages at school. Schools are required to make free drinking water available during mealtimes. However, evidence about the extent to which schools are meeting this requirement is mixed and suggests that there is more SFAs and schools can do to promote implementation of this requirement, maximize children’s access, and promote consumption of water at school [50,51,52]. 

Food manufacturers and parents have important roles to play in decreasing levels of added sugars in school meals and children’s diets. Food manufacturers should be encouraged to decrease added sugars in prepared foods and flavored milks developed specifically for school meal programs. Parents of school-age children should be encouraged to limit children’s intake of added sugars outside of school hours and in foods or meals students bring to school. Consistent with prior research [18], findings from this analysis suggest that these efforts should target, in addition to the foods noted above, sugar-sweetened beverages and candy. 

This analysis has several important strengths. It is the first analysis to examine levels of added sugars in school lunches and breakfasts using menu data from a nationally representative sample of schools operating under the nutrition standards that went into effect in SY 2012–2013. In addition to assessing the added sugars content of school meals and the percentage of schools meeting the DGA recommended limit for added sugars, the analysis identified the leading sources of added sugars in school meals. Findings from these analyses should be useful to USDA as they consider updating nutrition standards for school meals to incorporate goals for added sugars, in keeping with the 2015–2020 and 2020–2025 DGAs [2,53].

Nonetheless, the analysis has limitations. The analysis was not able to assess levels of added sugars in “competitive foods”—foods children may have access to outside of reimbursable school meals through a la carte sales in cafeterias during mealtimes, vending machines, school stores, snack bars, food carts/kiosks, or fundraisers [24]. Historically, these competitive foods have been high in added sugars and fats [38,54]. Schools have been working to improve the nutritional quality of competitive foods using USDA’s Smart Snacks in School standards, which took effect in SY 2014–2015 [55]. The SNMCS did not collect detailed data on the nutrient composition of competitive foods because the study’s design was finalized before the Smart Snacks standards were developed [24]. Depending on how well schools were complying with Smart Snacks standards, this analysis may overestimate amounts of added sugars consumed by children who included one or more competitive foods in the meals they consumed at school. To provide a comprehensive picture of students’ consumption of added sugars at school as well as schools’ compliance with the Smart Snacks standards, future studies should collect detailed data on the nutrient composition of competitive foods. In addition, findings from the analysis of children’s meal-specific dietary intakes should be considered descriptive only and interpreted with caution. The DGAs describe goals for an overall dietary pattern, which do not necessarily apply to individual meals. 

## Figures and Tables

**Table 1:** Added sugars in school breakfasts and lunches.

	School Breakfasts				School Lunches			
	Mean ± SE				Mean ± SE			
	*n*	Calories from Added Sugars	% of Calories from Added Sugars	% of Schools Exceeding DGA Limit	*n*	Calories from Added Sugars	% of Calories from Added Sugars	% of Schools Exceeding DGA Limit
All schools	1111	88 ± 2	17.0 ± 0.2	92.2 ± 1.3	1207	75 ± 1	11.2 ± 0.1	69.1 ± 2.1
School level								
Elementary school	415	84 ± 2	17.0 ± 0.3	90.8 ± 1.9	451	74 ± 1	11.5 ± 0.2	74.8 ± 2.6 ^a,b^
Middle school	352	93 ± 2	17.3 ± 0.3	93.9 ± 1.5	384	73 ± 1	11.0 ± 0.2	65.8 ± 2.9 ^c^
High school	344	92 ± 2	16.9 ± 0.3	94.5 ± 1.4	372	78 ± 2	10.6 ± 0.2	56.8 ± 3.5
School poverty level								
Low poverty	162	101 ± 4	17.7 ± 0.5	95.3 ± 2.1 ^d^	226	75 ± 2	11.1 ± 0.2	67.2 ± 4.0
Mid-low poverty	353	94 ± 3	17.7 ± 0.3	95.3 ± 1.6 ^e^	376	77 ± 2	11.3 ± 0.2	72.7 ± 3.5
Mid-high poverty	323	87 ± 3	17.0 ± 0.4	92.8 ± 2.2	330	76 ± 2	11.2 ± 0.2	66.3 ± 4.2
High poverty	273	77 ± 3	16.1 ± 0.5	87.3 ± 3.1	275	71 ± 2	11.0 ± 0.3	69.6 ± 4.0
School racial/ethnic composition								
Majority White	572	96 ± 2	17.8 ± 0.3	96.5 ± 0.9 ^f,g^	643	79 ± 1	11.5 ± 0.2	71.8 ± 2.8
Majority Black	88	84 ± 4	17.2 ± 0.7	90.2 ± 4.7	89	72 ± 3	11.1 ± 0.5	63.1 ± 8.0
Majority Hispanic	155	74 ± 3	15.4 ± 0.6	84.2 ± 4.9	156	66 ± 3	10.4 ± 0.4	63.5 ± 5.7
Diverse	288	80 ± 3	16.4 ± 0.5	88.5 ± 3.4	310	71 ± 1	10.9 ± 0.2	68.4 ± 4.1

**Notes:** The statistical significance of differences between subgroups in the percentage of schools exceeding the DGA limit was tested for all subgroups. Superscript letters indicate a statistically significant difference (*p* < 0.05) in the percentage of schools exceeding the DGA limit for the following subgroups: ^a^ Elementary versus middle. ^b^ Elementary versus high. ^c^ Middle versus high. ^d^ Low poverty versus high poverty. ^e^ Mid-low poverty versus high poverty. ^f^ Majority White versus majority Hispanic. ^g^ Majority White versus diverse. DGA: Dietary Guidelines for Americans; SE: standard error.

**Table 2:** Top ten sources of added sugars in school breakfasts and lunches.

School Breakfasts (*n* = 1111)	% Contribution to Total Amount	School Lunches (*n* = 1207)	% Contribution to Total Amount
Flavored skim milk	29.0	Flavored skim milk	46.9
Sweetened cold cereal	13.0	Condiments and toppings	9.0
Condiments and toppings	11.8	Flavored 1% milk	3.2
Muffins and sweet/quick breads	7.3	Breads, rolls, bagels, and other plain breads	2.7
Granola bars and breakfast bars	5.0	Canned peaches	2.4
Toaster pastries	4.3	Cookies, cakes, brownies	2.3
Pancakes, waffles, and French toast	3.8	Sandwich with breaded meat, poultry, or fish	2.1
Crackers, croutons, and pretzels	3.6	Juice	1.8
Cinnamon buns	2.8	Black, baked and other beans	1.8
Yogurt, low-fat/fat-free	2.8	Hamburgers and similar beef/pork sandwiches	1.8

**Note:** When data for the SNMCS were collected, the updated nutrition standards did not allow schools to offer flavored low-fat (1%) milk, but a small number of schools did not comply with this requirement.

**Table 3:** Added sugars in breakfasts and lunches consumed by school-age children on school days.

	Breakfast Intakes				Lunch Intakes			
	Mean ± SE				Mean ± SE			
	*n*	Calories from Added Sugars	% of Calories from Added Sugars	% of Children Exceeding DGA Limit	*n*	Calories from Added Sugars	% of Calories from Added Sugars	% of Children Exceeding DGA Limit
All students	1871	63 ± 2	16.1 ± 0.4	61.8 ± 1.5	2097	67 ± 3	11.0 ± 0.3	47.3 ± 1.5
School level								
Elementary school	694	61 ± 3	15.8 ± 0.6	63.4 ± 2.0	744	63 ± 4	11.3 ± 0.5	49.5 ± 2.4
Middle school	603	59 ± 3	16.0 ± 0.7	66.0 ± 3.0	683	59 ± 3	10.5 ± 0.5	45.1 ± 2.3
High school	574	68 ± 4	16.6 ± 0.9	56.8 ± 3.4	670	77 ± 4	10.9 ± 0.5	45.6 ± 2.6
Gender								
Female	902	62 ± 3	17.0 ± 0.6	63.9 ± 1.9	995	59 ± 3	10.4 ± 0.4	44.3 ± 2.0 ^a^
Male	955	64 ± 2	15.2 ± 0.5	59.8 ± 2.2	1079	75 ± 4	11.5 ± 0.4	50.2 ± 2.0
Eligible for free or reduced-price meals								
Yes	852	63 ± 3	15.6 ± 0.6	61.8 ± 2.2	967	62 ± 4	10.8 ± 0.5	50.0 ± 2.6
No	987	63 ± 3	16.6 ± 0.6	62.5 ± 1.8	1089	71 ± 3	11.1 ± 0.4	45.1 ± 2.1
Race/ethnicity								
Non-Hispanic White	840	65 ± 3	17.3 ± 0.6	63.6 ± 2.0 ^b^	927	74 ± 3	11.6 ± 0.4	50.8 ± 2.0 ^e,f^
Non-Hispanic Black	223	65 ± 6	17.1 ± 1.2	64.2 ± 3.9 ^c^	257	64 ± 7	10.8 ± 1.0	47.1 ± 4.4
Hispanic	479	61 ± 4	14.9 ± 0.6	63.2 ± 2.6 ^d^	537	58 ± 5	10.0 ± 0.6	43.6 ± 2.9
Multiracial/other	171	45 ± 5	11.4 ± 0.9	47.3 ± 4.4	181	62 ± 7	10.7 ± 1.0	41.5 ± 4.1
Participated in the National School Lunch Program								
Yes	1082	64 ± 3	15.7 ± 0.6	60.9 ± 1.9	1254	56 ± 3	10.2 ± 0.4	46.6 ± 1.9
No	789	62 ± 3	16.6 ± 0.7	63.0 ± 2.3	843	82 ± 4	12.1 ± 0.5	48.1 ± 2.3
Participated in the School Breakfast Program								
Yes	511	64 ± 4	15.8 ± 0.8	63.6 ± 3.6	504	55 ± 4	10.0 ± 0.6	48.2 ± 3.3
No	1360	62 ± 3	16.2 ± 0.5	61.2 ± 1.6	1593	70 ± 3	11.3 ± 0.4	47.0 ± 1.8

**Notes:** Samples exclude children who did not consume the associated meal. The participation variables reflect participation in the NSLP or SBP on the day covered in the 24-h recall. Superscript letters indicate a statistically significant difference (*p* < 0.05) in the percentage of children exceeding the DGA limit for the following subgroups: ^a^ Females versus males for lunches consumed. ^b^ Non-Hispanic White versus multiracial/other for breakfasts consumed. ^c^ Non-Hispanic Black versus multiracial/other for breakfasts consumed. ^d^ Hispanic versus multiracial/other for breakfasts consumed. ^e^ Non-Hispanic White versus Hispanic for lunches consumed. ^f^ Non-Hispanic White versus multiracial/other for lunches consumed. DGA: Dietary Guidelines for Americans; NSLP: National School Lunch Program; SBP: School Breakfast Program; SE: standard error.

**Table 4:** Top ten sources of added sugars in breakfasts and lunches consumed by school-age children on school days.

Breakfasts Consumed (*n* = 1871)	% Contribution to Total Amount	Lunches Consumed (*n* = 2097)	% Contribution to Total Amount
Sweetened cold cereal	23.0	Flavored skim milk	15.9
Condiments and toppings	12.8	Cake	11.3
Toaster pastries	4.9	Peanut butter sandwiches	6.8
Granola bars and breakfast bars	4.8	Candy	6.1
Tea and coffee	4.1	Fruit drinks	6.0
Fruit drinks	4.0	Condiments and toppings	5.9
Muffins and sweet/quick breads	3.9	Carbonated soda	4.5
Flavored skim milk	3.9	Sports and energy drinks	4.1
Carbonated soda	3.5	Tea and coffee	3.9
Yogurt, low-fat/fat-free	3.2	Granola bars and breakfast bars	3.6

**Table 5:** Added sugars in 24-h dietary intakes of school-age children on school days.

	*n*	24-h Intakes		
		Mean ± SE		
		Calories from Added Sugars	% of Calories from Added Sugars	% of Children Exceeding DGA Limit
All students	2165	263 ± 8	12.9 ± 0.3	63.2 ± 1.9
School level				
Elementary school	748	263 ± 13	13.1 ± 0.4	66.1 ± 2.7
Middle school	714	243 ± 10	12.8 ± 0.4	63.2 ± 2.2
High school	703	274 ± 12	12.7 ± 0.5	59.3 ± 3.1
Gender				
Female	1029	247 ± 9	13.0 ± 0.3	63.7 ± 2.1
Male	1113	279 ± 11	12.8 ± 0.4	62.6 ± 2.5
Eligible for free or reduced-price meals				
Yes	1004	267 ± 11	13.3 ± 0.4	64.7 ± 2.1
No	1119	260 ± 10	12.6 ± 0.4	62.0 ± 2.7
Race/ethnicity				
Non-Hispanic White	953	275 ± 12	13.5 ± 0.4	66.8 ± 2.6 ^a,b^
Non-Hispanic Black	267	293 ± 18	14.2 ± 0.6	70.7 ± 3.5 ^c,d^
Hispanic	551	234 ± 11	11.9 ± 0.4	58.6 ± 2.4
Multiracial/other	188	234 ± 21	11.0 ± 0.7	51.8 ± 4.6
Participated in the National School Lunch Program				
Yes	1254	264 ± 11	12.9 ± 0.4	63.8 ± 2.0
No	911	262 ± 10	12.9 ± 0.4	62.3 ± 2.6
Participated in the School Breakfast Program				
Yes	511	279 ± 12	13.2 ± 0.5	65.6 ± 3.0
No	1654	258 ± 10	12.8 ± 0.4	62.5 ± 2.2

**Notes:** The participation variables reflect participation in the NSLP or SBP on the day covered in the 24-h recall. Superscript letters indicate a statistically significant difference (*p* < 0.05) in the percentage of children exceeding the DGA limit for the following subgroups: ^a^ White versus Hispanic. ^b^ White versus multiracial/other. ^c^ Black versus Hispanic. ^d^ Black versus multiracial/other. DGA: Dietary Guidelines for Americans; NSLP: National School Lunch Program; SBP: School Breakfast Program; SE: standard error.

## Data Availability

The data used in this paper are restricted-use and come from a large, nationally representative study of the school meal programs that operate in the United States. Requests for access to the public use version of these data should be submitted via electronic mail to FNSStudies@usda.gov.

## References

[1] U.S. Department of Health and Human Services, U.S. Department of Agriculture (2015). Scientific Report of the 2015 Dietary Guidelines Advisory Committee: Advisory Report to the Secretary of Health and Human Services and the Secretary of Agriculture.

[2] U.S. Department of Health and Human Services, U.S. Department of Agriculture (2015). 2015–2020 Dietary Guidelines for Americans.

[3] Powell E.S., Smith-Taillie P.P., Popkin B.M. (2016). Added Sugars Intake Across the Distribution of Children and Adult Consumers: 1977–2012. J. Acad. Nutr. Diet..

[4] Te Morenga L., Mallard S., Mann J. (2012). Dietary Sugars and Body Weight: Systematic Review and Meta-Analyses of Randomised Controlled Trials and Cohort Studies. BMJ.

[5] Malik V.S., Pan A., Willett W.C., Hu F.B. (2013). Sugar-Sweetened Beverages and Weight Gain in Children and Adults: A Systematic Review and Meta-Analysis. Am. J. Clin. Nutr..

[6] Wang J.W., Shang L., Light K., O’Laughlin J., Paradis G., Gray-Donald K. (2015). Associations Between Added Sugar (Solid vs. liquid) Intakes, Diet Quality, and Adiposity Indicators in Canadian Children. Appl. Physiol. Nutr. Metab..

[7] De Ruyter J.C., Olthof M.R., Seidell J.C., Katan M.B. (2012). A Trial of Sugar-free or Sugar-Sweetened Beverages and Body Weight in Children. N. Engl. J. Med..

[8] Ebbeling C.B., Feldman H.A., Chomitz V.R., Antonelli T.A., Gortmaker S.L., Osganian S.K., Ludwig D.S. (2012). A Randomized Trial of Sugar-Sweetened Beverages and Adolescent Body Weight. N. Engl. J. Med..

[9] Louie J.C.Y., Tapsell L.C. (2015). Association Between Intake of Total vs Added Sugar on Diet Quality: A Systematic Review. Nutr. Rev..

[10] Moynihan P.J., Kelly S.A. (2014). Effect on Caries of Restricting Sugars Intake: Systematic Review to Inform WHO Guidelines. J. Dent. Res..

[11] Chi D.L., Scott J.M. (2019). Added Sugar and Dental Caries in Children: A Scientific Update and Future Steps. Dent. Clin. N. Am..

[12] Vos M.B., Kaar J.L., Welsh J.A., Van Horn L.V., Feig D.I., Anderson C.A., Patel M.J., Cruz Munos J., Krebs N.F., Xanthakos S.A. (2017). Added Sugars and Cardiovascular Disease Risk in Children: A Scientific Statement from the American Heart Association. Circulation.

[13] Lee A.K., Binongo J.N.G., Chowdhury R., Stein A.D., Gazmararian J.A., Vos M.B., Welsh J.A. (2014). Consumption of Less Than 10% of Total Energy From Added Sugars is Associated With Increasing HDL in Females During Adolescence: A Longitudinal Analysis. J. Am. Heart Assoc..

[14] National Heart Lung and Blood Institute (2012). Expert Panel on Integrated Guidelines for Cardiovascular Health and Risk Reduction in Children and Adolescents.

[15] De Moraes M.M., Mediano M.F.F., de Souza R.A.G., Moura A.S., da Veiga G.V., Sichieri R. (2017). Discouraging Soft Drink Consumption Reduces Blood Glucose and Cholesterol of Brazilian Elementary Students: Secondary Analysis of a Randomized Controlled Trial. Prev. Med..

[16] Bowman S. (2017). Added Sugars: Definition and Estimation in the USDA Food Patterns Equivalent Databases. J. Food Compost. Anal..

[17] World Health Organization (2015). Guideline: Sugars Intake for Adults and Children.

[18] Bowman S., Clemens J.C., Friday J.E., Schroeder N., LaComb R.P. (2019). Added Sugars in American Children’s Diet: What We Eat in America, NHANES 2015–2016.

[19] Food Research and Action Center National School Lunch Program. https://frac.org/programs/national-school-lunch-program.

[20] Forrestal S., Cabili C., Dotter D., Logan C.W., Connor P., Boyle M., Enver A., Nissar H. (2019). School Nutrition and Meal Cost Study, Final Report Volume 1: School Meal Program Operations and School Nutrition Environments.

[21] U.S. Department of Agriculture, Food and Nutrition Service National Level Annual Summary Tables: FY 1969–2019, National School Lunch: Participation and Meals Served. https://www.fns.usda.gov/pd/child-nutrition-tables.

[22] U.S. Department of Agriculture, Food and Nutrition Service National Level Annual Summary Tables: FY 1969–2020, School Breakfast: Participation and Meals Served. https://www.fns.usda.gov/pd/child-nutrition-tables.

[23] Cullen K.W., Chen T.-A. (2017). The Contribution of the USDA School Breakfast and Lunch Program Meals to Student Daily Dietary Intake. Prev. Med. Rep..

[24] Fox M.K., Gearan E., Cabili C., Dotter D., Niland K., Washburn L., Paxton N., Olsho L., LeClair L., Tran V. (2019). School Nutrition and Meal Cost Study, Final Report Volume 4: Student Participation, Satisfaction, Plate Waste, and Dietary Intakes.

[25] U.S. Department of Agriculture, Food and Nutrition Service (2012). Nutrition Standards in the National School Lunch and School Breakfast Programs: Final Rule. Fed. Regist..

[26] Gearan E., Fox M.K. (2020). Updated Nutrition Standards Have Significantly Improved the Nutritional Quality of School Lunches and Breakfasts. J. Acad. Nutr. Diet..

[27] Kinderknecht K., Harris C., Jones-Smith J. (2020). Association of the Healthy, Hunger-Free Kids Act with Dietary Quality among Children in the U.S. National School Lunch Program. JAMA.

[28] Au L.E., Rosen N.J., Fenton K., Hecht K., Ritchie L.D. (2016). Eating School Lunch is Associated with Higher Diet Quality among Elementary School Students. J. Acad. Nutr. Diet..

[29] Bergman E.A., Saade C., Shaw E., Englund T., Cashman L., Taylor K.W., Watkins T., Rushing K. (2014). Lunches Selected and Consumed from the National School Lunch Program in Schools Designated as Healthier U.S. School Challenge Schools are More Nutritious Than Lunches Brought from Home. J. Child Nutr. Manag..

[30] Au L.E., Gurzo K., Gosliner W., Webb K.L., Crawford P.B., Ritchie L.D. (2018). Eating School Meals Daily Is Associated with Healthier Dietary Intakes: The Healthy Communities Study. J. Acad. Nutr. Diet..

[31] Guenther P.M., Casavale K.O., Reedy J., Kirkpatrick S.I., Hiza H.A.B., Kuczynski K.J., Kahle L.L., Krebs-Smith S.M. (2013). Update of the Healthy Eating Index: HEI-2010. J. Acad. Nutr. Diet..

[32] U.S. Department of Agriculture, Center for Nutrition Policy and Promotion Healthy Eating Index-2010. CNPP Fact Sheet No. 2. https://www.fns.usda.gov/cnpp/healthy-eating-index-hei-reports.

[33] U.S. Department of Agriculture, U.S. Department of Health and Human Services (2010). Dietary Guidelines for Americans.

[34] Gearan E., Fox M.K., Niland K., Dotter D., Washburn L., Connor P., Olsho L., Wommack T. (2019). School Nutrition and Meal Cost Study Final Report Volume 2: Nutritional Characteristics of School Meals.

[35] Raper N., Perloff B., Ingwersen L., Steinfeldt L., Anand J. (2004). An Overview of USDA’s Dietary Intake Data System. J. Food Compos. Anal..

[36] U.S. Department of Agriculture, Agricultural Research Service (2014). USDA Food and Nutrient Database for Dietary Studies 2011–2012. https://www.ars.usda.gov/northeast-area/beltsville-mdbhnrc/beltsville-human-nutrition-research-center/food-surveys-research-group/docs/fndds/.

[37] U.S. Department of Agriculture, Agricultural Research Service (2014). Food Patterns Equivalents Database 2011–2012. https://www.ars.usda.gov/northeast-area/beltsville-md-bhnrc/beltsville-human-nutrition-research-center/food-surveys-research-group/docs/fped-overview/.

[38] Gordon A., Fox M.K., Clark M., Nogales R., Condon E., Gleason P., Sarin A. (2007). School Nutrition Dietary Assessment Study-III, Final Report Volume II: Student Participation and Dietary Intakes.

[39] Gleason P., Suitor C. (2001). Children’s Diets in the Mid-1990s: Dietary Intake and Its Relationship with School Meal Participation, CN01-CD1U.S.

[40] Zeidman E., Beyler N., Gearan E., Morrison N., Niland K., Washburn L., Carlson B. (2019). School Nutrition and Meal Cost Study: Study Design, Sampling, and Data Collection.

[41] U.S. Department of Education, National Center for Education Statistics (2011). 2010–2011 Common Core Data.

[42] O’Malley P.M., Johnston L.D., Delva J., Bachman J.G., Schulenberg J.E. (2007). Variation in Obesity Among American Secondary School Students by School and School Characteristics. Am. J. Prev. Med..

[43] Subar A.F., Krebs-Smith S.M., Cook A., Kahle L.L. (1998). Dietary Sources of Nutrients Among U.S. Children, 1989–1991. Pediatrics.

[44] Bardin S., Washburn L., Gearan E. (2020). Disparities in the Healthfulness of School Food Environments and the Nutritional Quality of School Lunches. Nutrients.

[45] U.S. Department of Agriculture, Food and Nutrition Service WIC Food Packages—Regulatory Requirements for WIC-Eligible Foods. https://www.fns.usda.gov/wic/wic-food-packages-regulatory-requirements-wic-eligible-foods.

[46] U.S. Food and Drug Administration Added Sugars: Now Listed on the Nutrition Facts Label. https://www.fda.gov/media/135299/download.

[47] U.S. Department of Agriculture, Food and Nutrition Service (2018). Child Nutrition Programs: Flexibilities for Milk, Whole Grains, and Sodium Requirements. Fed. Regist..

[48] Academy of Nutrition and Dietetics, Eat Right Pro Academy Comments to USDA re Child Nutrition Programs: Flexibilities for Milk, Whole Grains, and Sodium Requirements. https://www.eatrightpro.org/news-center/on-the-pulse-of-public-policy/regulatory-comments/comments-usda-child-nutrition-programs-flexibilities-milk-whole-grains-sodium-requirements.

[49] School Nutrition Association Preserve USDA’s 2018 Final Rule Child Nutrition Programs: Flexibilities for Milk, Whole Grains, and Sodium Requirements. https://schoolnutrition.org/uploadedFiles/Legislation_and_Policy/SNA_Policy_Resources/2020-Flexibility-Fact.pdf.

[50] Hood N.E., Turner L., Colabianchi N., Chaloupka F.J., Johnston L.D. (2014). Availability of Drinking Water in U.S. Public School Cafeterias. J. Acad. Nutr. Diet..

[51] Kenney E.L., Gortmaker S.L., Cohen J.F.W., Rimm E.B., Cradock A.L. (2016). Limited School Drinking Water Access for Youth. J. Adolesc. Health.

[52] Altman E.A., Lee K.L., Hecht C.A., Hampton K.E., Moreno G., Patel A.I. (2020). Drinking Water Access in California Schools: Room for Improvement Following Implementation of School Water Policies. Prev. Med. Rep..

[53] U.S. Department of Agriculture, U.S. Department of Health and Human Services (2020). Dietary Guidelines for Americans 2020–2025.

[54] Fox M.K., Gordon A., Nogales R., Wilson A. (2009). Availability and Consumption of Competitive Foods in U.S. Public Schools. J. Am. Diet. Assoc..

[55] U.S. Department of Agriculture, Food and Nutrition Service (2016). National School Lunch Program and School Breakfast Program: Nutrition Standards for All Foods Sold in School as Required by the Healthy, Hunger-Free Kids Act of 2010. Fed. Regist..

